# Molecular detection and genetic characteristics of *Babesia gibsoni* in dogs in Shaanxi Province, China

**DOI:** 10.1186/s13071-020-04232-w

**Published:** 2020-07-22

**Authors:** Wen-Ping Guo, Guang-Cheng Xie, Dan Li, Meng Su, Rui Jian, Luan-Ying Du

**Affiliations:** Department of Pathogenic Biology, College of Basic Medicine, Chengde Medical University, Chengde, Hebei China

**Keywords:** *B. gibsoni*, *18S* rRNA, ITS, *TRAP*, *cytb*, Genetic characteristic, Phylogenetic analysis

## Abstract

**Background:**

Several members of genus *Babesia* are important pathogens causing babesiosis in dogs. In China, at least five *Babesia* species have been described in dogs or ticks. This study sought to determine the prevalence and molecular characteristics of various *Babesia* spp. in dogs in cities in Shaanxi Province in China, including Xi’an and Hanzhong.

**Methods:**

A total of 371 blood samples were collected from pet dogs presenting to veterinary clinics in the cities of Xi’an and Hanzhong in Shaanxi, China. *Babesia* spp. DNA was detected *via* amplification of partial *18S* rRNA genes by semi-nested PCR. Almost full-length *18S* rRNA, ITS, partial *TRAP* and complete *cytb* genes were recovered for analysis of the genetic characteristics and relationships with known isolates.

**Results:**

A single species, *Babesia gibsoni*, was identified in dogs in Xi’an and Hanzhong. Consistently, *B. gibsoni* was also detected in 14 ticks collected from positive dogs. Sequence similarities and phylogenetic analysis suggested that the isolates identified herein showed a closer genetic relationship with isolates from East Asian countries rather than India, Bangladesh, or the USA. Sequence analysis based on tandem repeat analysis of the *TRAP* gene further revealed that specific haplotypes were circulating in both Xi’an and Hanzhong, with no specific regionality. In addition, 10.9% of all isolates with atovaquone (ATV)-resistance were identified because of M121I mutation in the deduced cytb protein.

**Conclusions:**

This study revealed a high prevalence rate of *Babesia* infection. *Babesia gibsoni* was the only *Babesia* species identified in cases of canine babesiosis in the cities of Xi’an and Hanzhong cities in Shaanxi, China. In addition, the *TRAP* gene presented high genetic diversity across isolates. Such information is useful for elucidating the epidemiological characteristics of canine babesiosis, as well as the overall genetic diversity of *Babesia* spp. circulating in dog populations in Shaanxi Province.
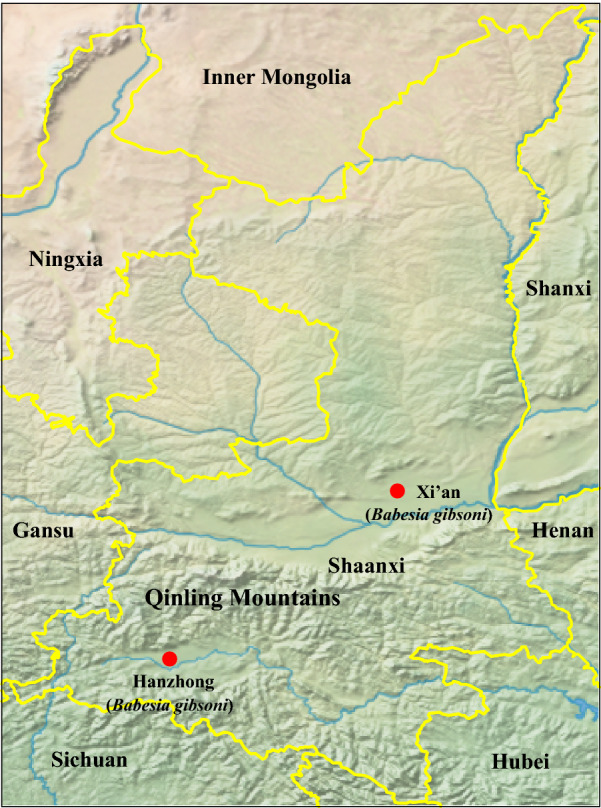

## Background

Babesiosis refers to a serious, worldwide, tick-borne hemoprotozoan disease caused by various *Babesia* spp. These *Babesia* spp. are classified as either large (3–5 μm) or small (1.5–2.5 μm) species based on their morphological size in erythrocytes [1]. Several members of the genus *Babesia* have gained increased attention due to their clinical significance in both veterinary and human medicine [2]. Canine babesiosis, a significant disease in dogs, is caused by at least seven validated *Babesia* species, including *B. gibsoni*, *B. conradae*, *B. vulpes*, *B. vogeli*, *B. canis*, *B. rossi* and *B. caballi* [3]. In addition, several unclassified *Babesia* spp. have also been detected in dogs [4–6]. Of these, *B. vogeli* and *B. gibsoni* present the most widespread distribution. *Babesia* spp. mainly spread between dogs through tick bites, although other ways of transmission, including dog bites, blood transfusions, and transplacental transmission is possible [7–9].

The clinical signs of canine babesiosis are similar for infection with both small and large *Babesia* spp. and include pyrexia, anemia, jaundice, hemoglobinuria, splenomegaly and weakness [3, 10–13]. The severity of canine babesiosis varies from mild to serious illness, largely depending on the specific species of infecting parasite, and conditions of the affected dog, such as age, nutritional and immune status [10–12]. For example, *B. rossi*, a highly virulent species, is associated with higher mortality in infected dogs compared to other *Babesia* species due to various complications of infection, such as disseminated intravascular coagulation and multi-organ dysfunction [14].

In China, serological investigation and molecular surveys of *Babesia* spp. infection have shown the geographical circulation of *B. gibsoni*, *B. caballi*, *B. canis*, *B. rossi* and *B. vogeli* in dog populations. As the most prevalent *Babesia* species, *B. gibsoni* is responsible for canine babesiosis in the central, southern and eastern regions of China, as well as Taiwan [15–20]. *Babesia vogeli* has been identified in dogs in Gansu, Jiangxi, Guangdong and Hunan provinces [21, 22] and in ticks in Henan [23]. *Babesia canis* has been reported in dogs in Henan [24] and various *18S* rRNA gene sequences of *B. canis* have been identified in dogs and ticks in Hunan and Qinghai provinces, respectively. *Babesia rossi* isolated identified in dogs in Hunan are further available in the GenBank database. *Babesia caballi* has been detected in ticks in the Xinjiang Uygur autonomous region and Jilin Province, as well as in horses (rather than dogs) in Gansu Province [25, 26]. Importantly, as a zoonotic agent, *B. caballi* has also been detected in one human blood sample (GenBank: KJ715182).

To date, no information is available regarding the prevalence of babesiosis in dogs in Shaanxi Province in China. To better understand the prevalence of various *Babesia* spp. in dogs, blood samples were collected in the cities of Xi’an and Hanzhong to characterize the prevalence and genetic diversity of *Babesia* spp. infections in dogs in Shaanxi Province.

## Methods

### Collection of canine blood samples and ticks

**Fig. 1:**
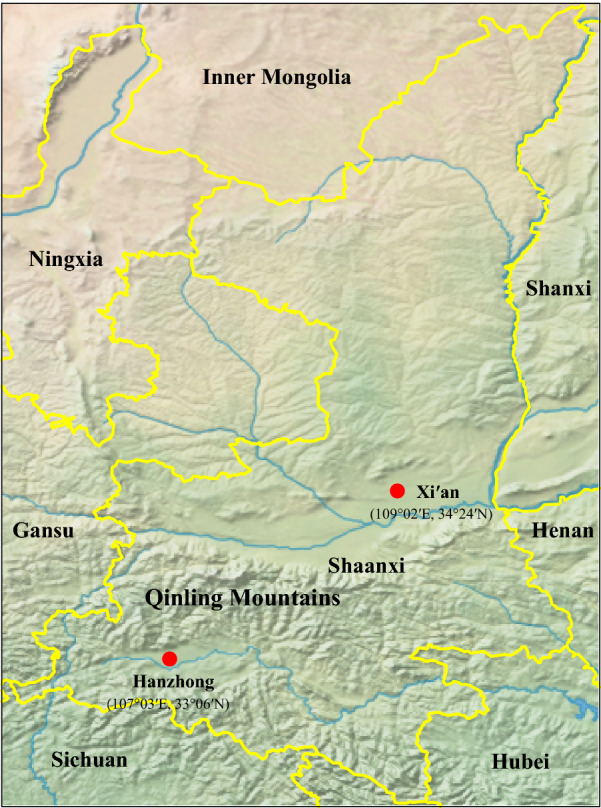
Geographic distribution of canine blood samples used for *Babesia* spp. detection in Xi’an and Hanzhong cities in the Shaanxi Province of China

Xi’an city is located in the middle of the Guanzhong Plain with the Qinling Mountains to the south, and its average elevation is 400 meters above sea level. Xi’an is characterized by a semi-moist monsoon climate with a clear distinction between the four seasons, and the annual average temperature is approximately 13 °C. Hanzhong city is located in the Hanjiang River basin with the Qinling Mountains to the north, and its average elevation is 550 meters above sea level. Hanzhong is characterized by a subtropical moist monsoon climate, with the annual average temperature of approximately 14 °C. From 2017 to 2018, EDTA-anticoagulated whole blood samples were aseptically collected from 371 pet dogs at four veterinary clinics located in urban areas, including two in Xi’an city and two in Hanzhong city in Shaanxi Province, China (Fig. 1). Among these, 260 (144 from Hanzhong and 116 from Xi’an) whole blood samples were collected from dogs suspected of having babesiosis that presented with at least two of the following clinical signs: fever; jaundice; anemia; thrombocytopenia; or hemoglobinuria. Moreover, all babesiosis-suspected dogs had a history of outdoor activities in the field and tick bites based on their owners’ statements. Additionally, 111 apparently healthy dogs (64 from Xi’an and 47 from Hanzhong) attended for prophylactic procedures were randomly selected, none of which presented with any of the above-mentioned clinical signs based on their owners’ statements. Furthermore, 15 adult and 2 nymphal ticks were collected from 9 babesiosis-suspected dogs. The tick species were first identified morphologically [27], and then further confirmed by analysis of the 710 bp *cox*1 gene [28].

### DNA extraction and detection of *Babesia*

Total DNA was extracted from canine blood samples using an E.Z.N.A.^®^ Blood DNA Kit (Omega, Norcross, GA, USA) according to the manufacturer’s recommendations. Meanwhile, total DNA was also extracted individually from all ticks using the E.Z.N.A.^®^ Tissue DNA Kit (Omega) according to the manufacturer’s instructions.

*Babesia* spp. DNA was detected using primers targeting the *18S* ribosomal RNA (rRNA) gene using a semi-nested polymerase chain reaction (PCR) [29]. Primary primer sets BS1 and PiroC, and secondary PiroA and PiroC, were used to amplify a 352-bp *18S* rRNA gene fragment (Table 1).

**Table 1:** Primers used in this study

Target gene	Primer name	Oligonucleotide sequence (5’-3’)	Product size (bp)	References
Partial *rrs*	BS1	GACGGTAGGGTATTGGCCT	380	[29]
	PiroA	ATTACCCAATCCTGACACAGGG		
	PiroC	CCAACAAAATAGAACCAAAGTCCTAC		
Nearly complete *rrs*	P1	AACCTGGTTGATCCTGCCAGTAGTCAT	1700	[15]
	P2	GAT CCT TCT GCA GGT TCA CCT AC		
Partial ITS	ITS F	GAGAAGTCGTAACAAGGTTTCCG	1100	[15]
	ITS 2	ACAATTTGCGTTCAATCCCA		
*Cytb*	Bgcytb-F	AGTGAAGGAAYTTGACAGGT	1200	This study
	Bgcytb-R	CTTTCCTATTCCTTACGTAC		
Partial *TRAP*	BgTRAPtF1	GTGACACTACAACGTTGTCTG	1100	[30]
	BgTRAPtR1	TGTTGATCCTCGTACAGTCC		
	BgTRAPtF2	GGTTAACAGTGGTTCTGTGAG		
	BgTRAPtR2	CTGGCGTCCATATCATAGTC		

### Amplification of nearly complete *18S* rRNA gene, ITS region, partial *TRAP* gene and complete *cytb* gene

A nearly complete *18S* rRNA gene, ITS region, partial thrombospondin-related adhesive protein (*TRAP*) gene and complete *cytb* gene were recovered from positive samples to investigate the genetic relationships among *Babesia* species. All the primer sequences were shown in Table 1 in detail. The nearly full length *18S* rRNA gene and the ITS region were amplified using the primer pairs P1/P2 and ITSF/ITS2, respectively, as previously described [15]. The 1100 bp *TRAP* gene was amplified by nested PCR using external primer set BgTRAPtF1 and BgTRAPtR1 and internal primer set BgTRAPtF2 and BgTRAPtR2 [30].

The complete *cytb* gene was amplified with primer set Bgcytb-F and Bgcytb-R designed in-house based on the conserved regions of *cytb* gene sequences of the genus *Babesia* available in the GenBank database. PCR was performed in a 50 μl volume including 25 μl Premix *Taq* (Takara), 3μl extracted total DNA as a template, 2 μl of each primer (10 pmol) and 18 μl water. The PCR cycling condition comprised an initial denaturation at 94 °C for 5 min, followed by 35 cycles denaturation at 94 °C for 40 s, annealing at 50 °C for 40 s, and elongation at 72 °C for 1 min, with a final extension step at 72 °C for 7 min.

### Cloning and sequencing of PCR products

All PCR amplicons were electrophoresed in 1.0% agarose gels, and those with expected size were purified using a Gel Extraction kit (TaKaRa). Partial *18S* rRNA gene sequences were subjected to sequencing in both directions using an Applied Biosystems 3130 Genetic Analyzer with a BigDye v3.1 Terminator cycle sequencing kit (Applied Biosystems Inc., Carlsbad, CA, USA). The purified PCR products were cloned into pMD19-T vector (TaKaRa, Dalian, China), and then the recombinant plasmid was transformed into *Escherichia coli* DH5α competent cells. Positive clones were identified by PCR and at least 3 positive clones were sequenced for each PCR product to determine a consensus sequence.

### Sequence and phylogenetic analyses

DNA sequences of the *18S* rRNA, ITS, *TRAP* and *cytb* genes recovered from positive samples were assembled and edited using the Lasergene program (DNASTAR, Inc., Madison, WI, USA). The obtained sequences were subjected to BLAST analysis against the GenBank database. The nucleotide identity was calculated using the ClustalW method implemented in Lasergene. The alignment among the new sequences obtained here and the reference sequences retrieved from GenBank was conducted using MAFFT (version 7) [31]. The optimal nucleotide substitution model, GTR+Γ+I, was determined using jModeltest 0.1 [32]. Phylogenetic trees were reconstructed using the Maximum Likelihood (ML) method based on the four above-mentioned 4 gene sequences using MEGA7 [33], with 1000 replications for bootstrap test.

### Tandem repeat analysis of *TRAP* gene fragments

Tandem Repeats Finder (version 4.09; http://tandem.bu.edu/trf/trf.html), a program for the detection of tandem repeats (TR) in a DNA sequence, was used to find out the location and to display TR DNA sequences in *TRAP* gene fragments, as previously described [34, 35].

## Results

### Detection and identification of *Babesia* spp

Of the 260 babesiosis-suspected dogs, PCR products with expected size were successfully amplified from 73 and 94 samples collected in Xi’an and Hanzhong, respectively. Sequencing and further BLAST analysis showed that all sequences had the highest nucleotide identity with sequences of *B. gibsoni* (more than 99.0%), suggesting *B. gibsoni* infection in all babesiosis-suspected dogs. The overall prevalence of *B. gibsoni* in babesiosis-suspected dogs was 64.2% (62.9% in Xi’an and 65.3% in Hanzhong, respectively; Table 2). Meanwhile, *B. gibsoni* was also detected in 4.7% and 10.6% of healthy dog samples collected in Xi’an (3/64) and Hanzhong (5/47), respectively, with an overall infection rate of 7.2% in healthy dogs. The positive rates of *B. gibsoni* infection in relation to season, age, sex, and breed of dogs are shown in Table 3.

**Table 2:** Prevalence of *B. gibsoni* in dogs in Xi’an and Hanzhong cities, China

Regions	Health condition	No. of blood samples	No. of positive blood samples	Positive rate (%)
Xi’an	Babesiosis-suspected	116	73	62.9
	healthy	64	3	4.7
Hanzhong	Babesiosis-suspected	144	94	65.3
	healthy	47	5	10.6

**Table 3:** *Babesia gibsoni* infections broken down by season, age, sex and breed of dogs in Xi’an and Hanzhong cities, China

Factor	Parameter	No. of positive blood samples	Positive rate (%)^a^
Season	Spring	19	10.9
	Summer	56	32.0
	Autumn	65	37.1
	Winter	35	20.0
Age	0–1 years-old	27	15.4
	1–3 years-old	82	46.9
	> 3 years-old	66	37.7
Sex	Male	92	52.6
	Female	83	47.4
Breed	Poodle	78	44.6
	Pomeranian	37	21.1
	Golden Retriever	20	11.4
	Mongrel dog	19	10.9
	Labrador	10	5.7
	Others	11	6.3

^a^ No. of positive blood samples /total positive number (*n* = 175)

All the infested ticks were identified as *Haemaphysalis longicornis* based on their morphological characters and the position of their *cox*1 gene sequences in the phylogenetic tree (Additional file 1: Figure S1). Consistent with the finding that 8 out of 9 dogs infested with ticks tested positive for *B. gibsoni*, 14 ticks collected from these 8 dogs also tested positive. In contrast, 3 other ticks sampled from a single *B. gibsoni*-negative dog also tested negative.

### Genetic variation and phylogenetic analysis of the *18S* rRNA gene and ITS region

Nearly full-length *18S* rRNA gene sequences of *B. gibsoni* obtained from all positive samples (MN928814-MN928833) shared 99.3–100% nucleotide identity with each other, as well as a high identity of 99.0–100% with known *18S* rRNA gene sequences. They specifically showed 100% nucleotide identity with Shikoku 1 (GenBank: AB478329), Aomori (GenBank: AB118032), and Okinawa (GenBank: AB478328) from Japan, Jeju 4 (GenBank: AB478323) from South Korea and TWN5 (GenBank: JQ710685) from Taiwan. Partial *18S* rRNA gene sequences recovered from tick samples were identical to those obtained from the positive canine blood samples. The 1100 bp ITS sequences recovered from all the positive samples (MN928834-MN928851) were also closely related, with less than 0.2% divergence from each other, and less than 1% divergence from known ITS sequences.

**Fig. 2:**
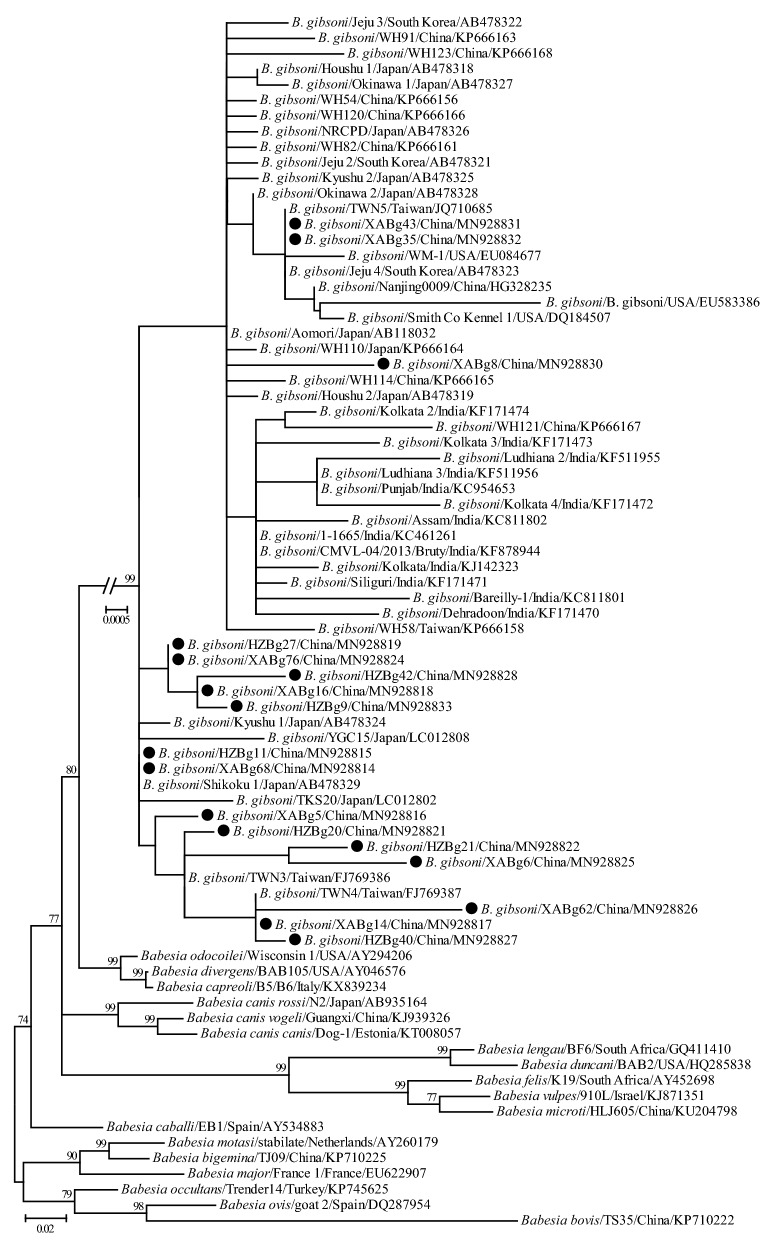
Phylogenetic tree based on *18S* rRNA gene sequences of *B. gibsoni* indicating genetic relationships between the new sequences obtained in this study and known sequences. Numbers at each node indicate bootstrap values (only numbers > 70 are shown). The tree was mid-point rooted for clarity, and the scale-bar represents the number of nucleotide substitutions per site. Representative strains were used to reconstruct the tree and marked by circles

**Fig. 3:**
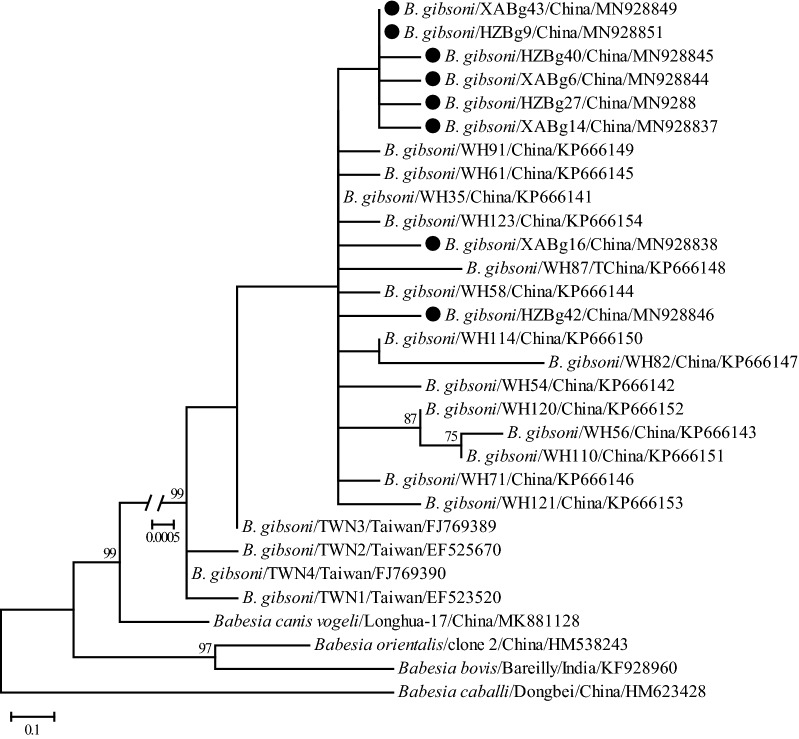
Phylogenetic tree based on ITS sequences of *B. gibsoni* indicating genetic relationship between the new sequences obtained in this study and known sequences. Numbers at each node indicate bootstrap values (only numbers > 70 are shown). The tree was mid-point rooted for clarity, and the scale-bar represents the number of nucleotide substitutions per site. Representative strains were used to reconstruct the tree and marked by circles

Phylogenetic trees were reconstructed based on representative *18S* rRNA and ITS sequences (only 1 of the sequences with 100% identity was included from each city, except XABg43 and XABg35) using the ML method. Both *18S* rRNA (> 1200 bp) and ITS (> 900 bp) sequences deposited on GenBank and those determined in the present study were used to perform phylogenetic analysis. The *18S* rRNA tree was low-resolution since it was highly conserved among different variants of *B. gibsoni*. Generally, all *18S* rRNA sequences were divided into two clades, albeit not well supported (Fig. 2). Three isolates recovered in this study were located in the first clade, including two (XABg43 and XABg35) clustering with those from Nanjing, Taiwan, Japan and the USA, and one (XABg8) clustered with those from Taiwan and Japan. All others were located in another clade and had a close phylogenetic relationship with canine isolates detected in Wuhan and Japan. In addition, the representative ITS sequences (only one of the sequences with 100% identity was included) in this study clustered together and had a closer phylogenetic relationship with sequences isolated from dogs in Wuhan and Taiwan, rather than India and the USA (Fig. 3).

### Genetic variation and phylogenetic analysis of the *TRAP* gene

The *TRAP* gene (1100 bp) fragment was recovered from all *B. gibsoni* isolates; all sequences (MN928852-MN928879) presented 98.4–100% nucleotide identity and 95.6–100% amino acid identity with each other. When compared with known *TRAP* sequences of *B. gibsoni*, they showed 86.8–99.8% nucleotide identity and 73.3–100% amino acid identity. Sequence comparison of *TRAP* sequences between those obtained here and other known relevant complete sequences showed that amino acid residues between 265 and 431 of the TRAP protein, including both partial von Willebrand Factor-like A (vWF-like A) and thrombospondin type I repeat (TSR) domains, were well-conserved (Additional file 2: Figure S2). Amino acid mutations primarily occurred after the TSR domain, and a large fragment deletion was observed at position 449–495, similar to isolates Kansai 59 (GenBank: KR013043), BgTRAP-Shikoku 2 (GenBank: AB478349), BgTRAP-Houshu 1 (GenBank: AB478341), BgTRAP-Houshu 2 (GenBank: AB478342), BgTRAP-Jeju 1 (GenBank: AB478343) and TWN6 (GenBank: JN247443). Amino acid mutations occurred in the transmembrane region of 7 sequences, mainly from Val (V) to Glu (E) and Tyr (Y) to Asp (D).

**Fig. 4:**
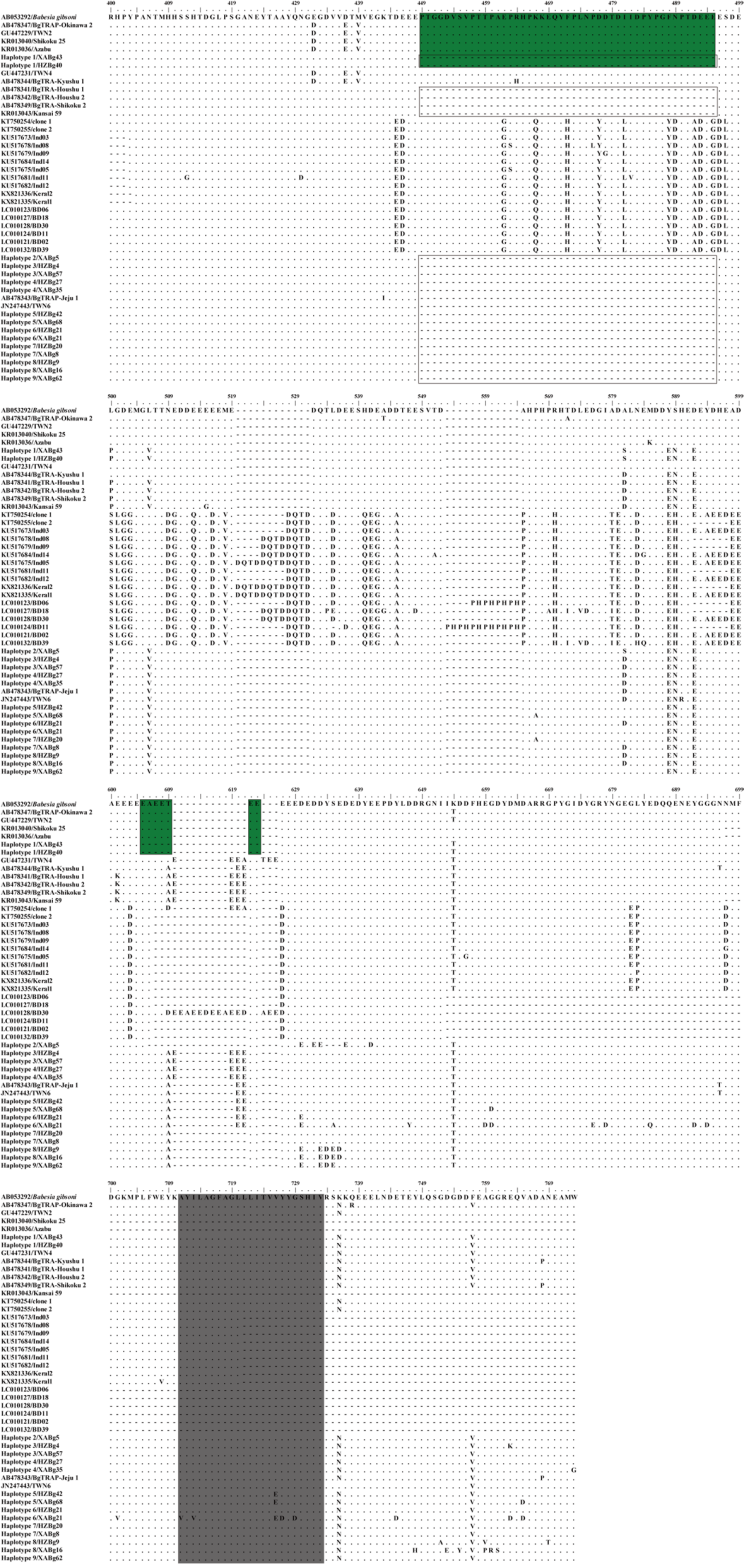
Alignment of representative TRAP amino acid sequences after the TSR region in each haplotype recovered in this study alongside samples in the GenBank database. A large fragment deletion was identified (box). Haplotype 1 identified in this study showed different insertion/deletion patterns compared to several other East Asian isolates from Japan, Korea and Taiwan (indicated in green). The transmembrane region is indicated in gray

Comparative analysis of the deduced amino acid sequences revealed the presence of 9 different haplotypes of TRAP based on tandem repeat analysis. More specifically, haplotypes 1 and 2, 3 and 4, 5 and 6, and 7, 8, and 9 had the same insertion/deletion pattern after the TSR region, respectively (Fig. 4). In both cities, all of these haplotypes were identified, with haplotypes 3 and 5 being the predominant ones. In addition to 59 isolates identified in this study, haplotype 5 also included BgTRAP-Jeju 1 (GenBank: AB478343) from South Korea and TWN6 (GenBank: JN247443) from Taiwan (Fig. 4). Although no tandem repeats were found in the consensus patterns, haplotype 1 identified in this study presented different insertion/deletion patterns compared to several other East Asian isolates from Japan, Korea and Taiwan (Fig. 4). TR analysis of the partial *TRAP* gene revealed 0 to 5 repeats in Xi’an and Hanzhong isolates (Table 4). Four consensus patterns (GGA GGA, GAG GAA GAG GAA, GGA GGA GGA GGA AGA GGA AGA and GCG GAG GAA GAG GAA GAG GAA GAG GAG) were common to most isolates found in Shaanxi Province, China.

**Table 4:** Tandem repeat analysis of TRAP gene fragments

Haplotype	No. of positive blood samples	No. of repeats	Position	Consensus pattern	Consensus size (bp)	No. of copy	Percent match
1	28 (*N*_Xi’an_ = 17, *N*_Hanzhong_ = 11)	–	–	–	–	–	–
2	3 (*N*_Xi’an_ = 1, *N*_Hanzhong_ = 2)	3	795–845	GGAGGA	6	8.5	93
			796–845	GAGGAAGAGGAA	12	3.9	82
			795–845	GGAGGAGGAGGAAGAGGAAGA	21	2.4	96
3	42 (*N*_Xi’an_ = 19, *N*_Hanzhong_ = 23)	5	796–857	GAGGAAGAGGAA	12	4.9	77
			796–857	GAGGAGGAGGAAGAGGAA	18	3.3	84
			795–857	GGAGGAGGAGGAAGAGGAAGA	21	3.1	84
			793–845	GCGGAGGAAGAGGAAGAGGAAGAGGAG	27	2.0	92
			823–857	GAGGAA	6	5.8	96
4	6 (*N*_Xi’an_ = 2, *N*_Hanzhong_ = 4	3	795–845	GGAGGAGGAAGAAGAGGAAGA	21	2.6	81
			793–845	GCGGAGGAAGAAGAAGAGGAAGAGGAG	27	2.0	88
			823–866	GAGGAA	6	5.8	96
5	59 (*N*_Xi’an_ = 36, *N*_Hanzhong_ = 23)	2	795–845	GGAGGAGGAGGAAGAGGAAGA	21	2.4	86
			793–845	GCGGAGGAAGAGGAAGAGGAAGAGGAG	27	2.0	92
6	6 (*N*_Xi’an_ = 5, *N*_Hanzhong_ = 1)	4	796–851	GAGGAAGAGGAA	12	4.4	76
			796–851	GAGGAGGAGGAAGAGGAA	18	2.9	84
			795–851	GGAGGAGGAGGAAGAGGAAGA	21	2.9	84
			793–845	GCGGAGGAAGAGGAAGAGGAAGAGGAG	27	2.0	92
7	22 (*N*_Xi’an_ = 17, *N*_Hanzhong_ = 5)	1	795–845	GGAGGAGGAGGAAGAGGAAGA	21	2.4	86
8	6 (*N*_Xi’an_ = 3, *N*_Hanzhong_ = 3)	4	795–845	GGAGGAGGAGGAAGAGGAAGA	21	2.4	86
			793–845	GCGGAGGAAGAGGAAGAGGAAGAGGAG	27	2.0	92
			823–866	GAGGAAGAGGAT	12	3.7	87
			823–857	GAGGAA	6	5.8	96
9	3 (*N*_Xi’an_ = 2, *N*_Hanzhong_ = 1)	3	795–845	GGAGGAGGAGGAAGAGGAAGA	21	2.4	86
			793–845	GCGGAGGAAGAGGAAGAGGAAGAGGAG	27	2.0	92
			823–866	GAGGAA	6	7.3	89

–, no repeats found

**Fig. 5:**
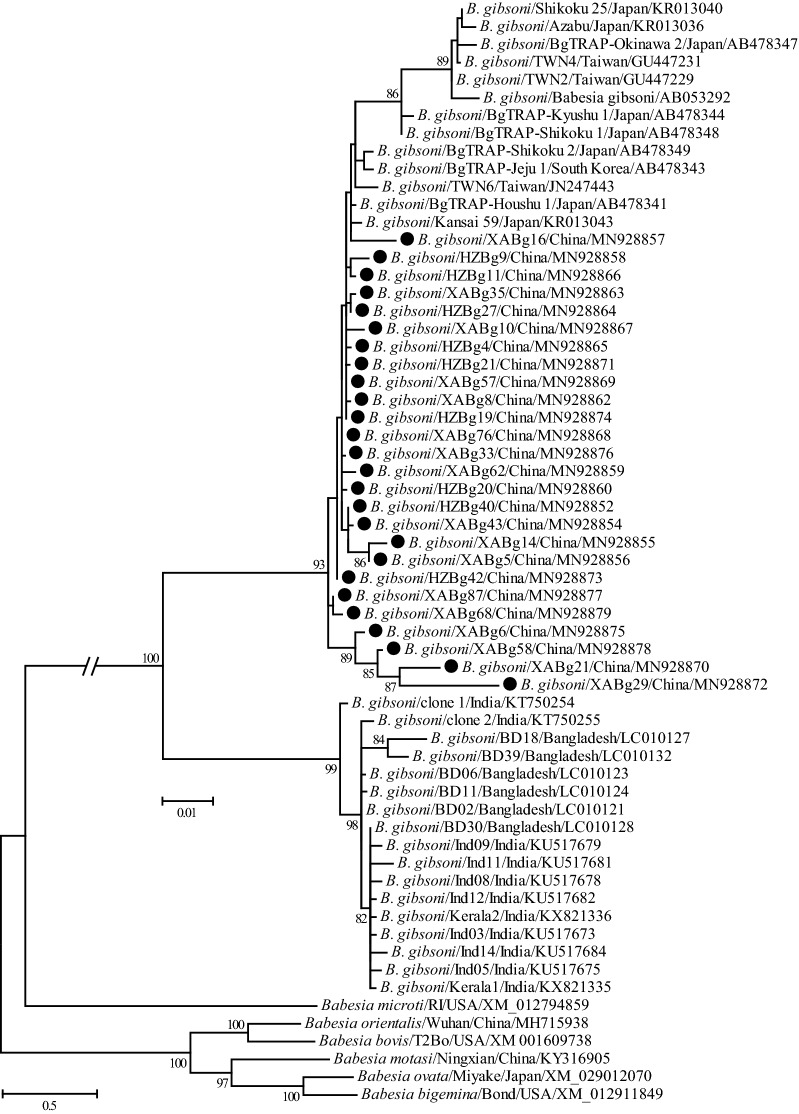
Phylogenetic tree based on *TRAP* gene sequences of *B. gibsoni* indicating genetic relationship between the new sequences obtained in this study and known sequences. Numbers at each node indicate bootstrap values (only numbers > 70 are shown). The tree was mid-point rooted for clarity, and the scale-bar represents the number of nucleotide substitutions per site. Representative strains were used to reconstruct the tree and marked by circles

The representatives (only one of the sequences with 100% identity was included in each city) of partial *TRAP* gene fragments were used to reconstruct a phylogenetic tree using the ML method. The results revealed that all *B. gibsoni* isolates were classified into two well-supported clusters (Fig. 5). The isolates identified in this study, as well as those from Taiwan, South Korea, and Japan formed the first cluster, and those identified in India and Bangladesh formed the second cluster. In the first cluster, the Shaanxi isolates had the closest relationship with BgTRAP-Jeju 1, BgTRAP-Houshu 1, BgTRAP-Houshu 2, BgTRAP-Shikoku 2, Kansai 59 and TWN6, followed by other Asian *B. gibsoni* isolates, including Taiwan, South Korea and Japan; they were more distantly related to isolates from India and Bangladesh.

### Genetic variation and phylogenetic analysis of the *cytb* gene

One hundred and seventy-five full length *cytb* gene sequences were obtained from *B. gibsoni-*positive samples (MN928880 to MN928897), which presented 99.5–100% nucleotide identity and 99.2–100% amino acid identity with each other. In addition, these sequences also shared 99.0–100% nucleotide and 99.2–100% amino acid identity with known *cytb* gene sequences of *B. gibsoni*, especially WH58 (GenBank: KP666169) from the city of Wuhan in China. Comparative analysis of the deduced amino acid sequences revealed that amino acid residue Ile at position 121 was present in 19 isolates (19/175, 10.9%), which is responsible for the atovaquone (ATV)-resistant phenotype confirmed in previous studies [36, 37]. However, Ile and Val were not identified at positions 220 and 303, respectively, which have also been associated with ATV-resistance.

**Fig. 6:**
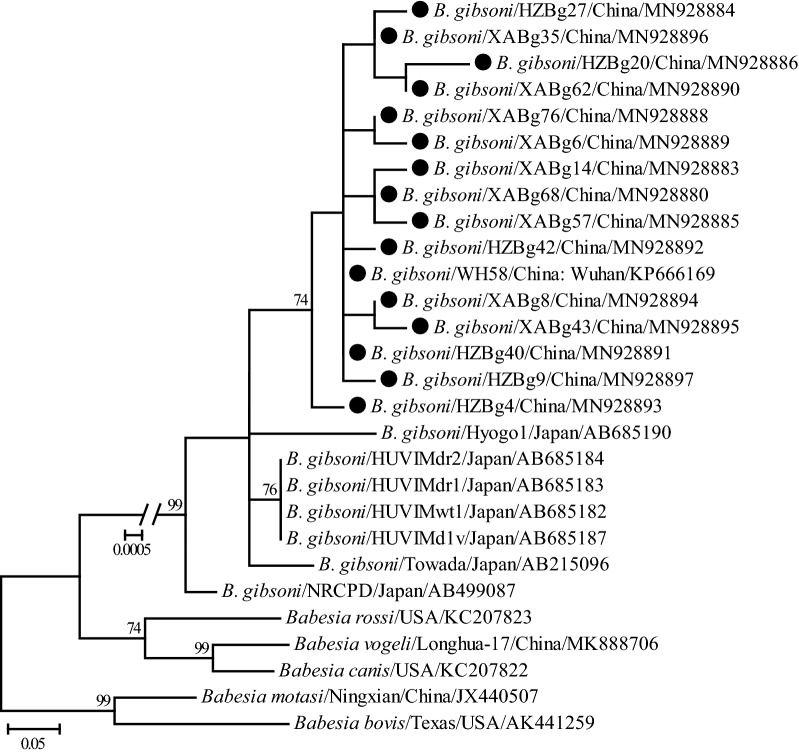
Phylogenetic tree based on *cytb* gene sequences of *B. gibsoni* indicating the genetic relationship between the new sequences obtained in this study and known sequences. Numbers at each node indicate bootstrap values (only numbers > 70 are shown). The tree was mid-point rooted for clarity, and the scale-bar represents the number of nucleotide substitutions per site. Representative strains were used to reconstruct the tree and shown in bold

Consistent with the nucleotide identity analysis, the representative *cytb* gene sequences (only one of the sequences with 100% identity was included in each city) of *B. gibsoni*, including these determined in the present study, demonstrated a close phylogenetic relationship with each other due to shorter genetic distances shared among them (Fig. 6). Generally, the *cytb* gene sequences recovered in this study had the closest relationship with isolate WH58 (GenBank: KP666169) from Wuhan, China, rather than other isolates from Japan.

## Discussion

Tick-borne *Babesia* spp. infections in dogs continue to increase worldwide, primarily in many developing countries but also in several developed countries. Previous epidemiological studies regarding *Babesia* infection in dogs and ticks demonstrated that four species of *Babesia* were circulating in China, and *B. gibsoni* was the predominant pathogen causing babesiosis in the central, southern and eastern regions of China [18–20, 22, 23, 38]. However, little information concerning the epidemiology of *Babesia* spp. in dogs in Shaanxi has been available. The results of an investigation of the prevalence of tick-borne pathogens in ten provinces of China showed that no *B. gibsoni* DNA was detected in 56 canine blood samples collected from Yangling in Shaanxi Province [19]. Nevertheless, another study reported the presence of *B. gibsoni* by detecting antibodies specific against the TRAP protein, with a positive rate of 8.33% in Xi’an in Shaanxi Province [39]. In the present study, molecular epidemiological analysis of *Babesia* infection in dogs was performed in Xi’an and Hanzhong, two cities in Shaanxi Province. The results suggest that *B. gibsoni* is the only *Babesia* species infecting all positive samples, as well as ticks collected from dogs positive for *B. gibsoni* infection. Taken together, these data indicate that the *B. gibsoni* parasite is widely distributed in China, although further investigations are warranted.

In the present study, the prevalence rate of *B. gibsoni* infection in babesiosis-suspected dogs was 64.2% in Xi’an and Hanzhong. Interestingly, 7.2% of healthy dogs were also positive for *B. gibsoni*, consistent with a prior report of *B. vogeli* infection in healthy pet dogs in Shenzhen, China [40]. These results suggest that both Xi’an and Hanzhong are severely affected by *B. gibsoni*. In terms of season and age of dogs, our results were consistent with those reported from India [41] and Australia [42], although they are in contrast to those from Jiangxi Province in China [22]. In this study, babesiosis-suspected cases were confirmed by detecting the DNA of various *Babesia* spp., strengthening the reliability of the prevalence rate identified for *B. gibsoni* in different season, age, sex and breed of dogs, as cases initially presenting with babesiosis-like symptoms may be infected by other known pathogens, such as Rickettsiales, *Hepatozoon* spp., *Theileria* spp., *Dirofilaria* spp., and *Leishmania* spp. [43–47], as well as unknown pathogens, and even non-infectious factors.

Similarity and phylogenetic analyses revealed that *18S* rRNA and ITS genes of *B. gibsoni* isolates identified in this study were highly conserved, and shared a closer relationship with those identified in Japan, Taiwan, Korea, and other parts of China, and were more distantly related to those in India, Bangladesh and the USA [30, 48], suggesting that geographic clustering pattern exists to a certain extent. Phylogenetic and similarity analyses based on the *TRAP* gene revealed a high genetic diversity of *B. gibsoni* isolates identified in this study, which may suggest that having a diverse TRAP protein may be favorable for pathogens to escape the host immune system [49]. All the isolates identified in this study were generally more closely related to each other and those detected in Japan, Taiwan, Korea, and other parts of China, while they were more distant to those in India, Bangladesh and the USA. Similarity and phylogenetic analyses based on the *cytb* gene suggest that *B. gibsoni* isolates determined in this study were closely related to those previously identified from Japan, although they formed two lineages based on regionality.

The *TRAP* gene is one of the more highly immunogenic proteins in *B. gibsoni* and possesses function in the parasite’s motility and invasion of red blood cells [50, 51]. This protein contains three domains: a vWF-like A domain; a TSR domain; and a short acidic cytoplasmic tail domain (CTD) [50, 51]. Consistent with previous studies, the vWF-like A and TSR domains were conserved in all isolates identified in this study, which have been used to develop a serological diagnostic assay and a vaccine candidate [52, 53]. However, regions between the TSR and the CTD present high genetic polymorphisms, including a large fragment deletion, which are suitable for genetic diversity and phylogenetic analysis of different *B. gibsoni* isolates [17, 54]. Moreover, amino acid mutations in the transmembrane region of 11 sequences were identified that have not been reported in previous studies, and the mechanism(s) by which these amino acid mutations may affect the function of TRAP protein require further investigation. TR analysis identified nine different haplotypes circulating in Xi’an and Hanzhong; all haplotypes were detected in both cities, suggesting no specific distribution pattern although Xi’an and Hanzhong are located on the north and south sides of the Qinling Mountains, respectively.

To our knowledge, few studies have focused on analyzing the *cytb* gene of *Babesia*. However, the *cytb* gene of *B. gibsoni* has gained increasing attention because amino acid residue Ile at position 121 has been associated with an ATV-resistant phenotype [36, 37]. To date, only one *cytb* sequence of *B. gibsoni* identified from China has been available in the GenBank database. To the best of our knowledge, our study is the first report concerning an AA substitution in the *cytb* gene of *B. gibsoni* related to ATV-resistance. Our results showed that M121I was observed in the deduced amino acid sequences recovered from canine blood samples in both Xi’an and Hanzhong, revealing that *B. gibsoni* with ATV-resistance has been naturally circulating in China. In the present study, its prevalence reached 10.9%, which is higher than it in Japan [55]. Interestingly, AA mutations V220I or I303V, which may also be linked to the ATV-resistance [37], were not identified in this study.

## Conclusions

Our findings indicate that *B. gibsoni* is the causative agent responsible of canine babesiosis in Xi’an and Hanzhong cities in the Shaanxi Province of China. Its high prevalence rate reveals a possibly serious epidemic in local dog populations. In addition, substantial great genetic diversity was observed based on analysis of the partial *TRAP* gene, and an AA mutation associated with ATV-resistance was confirmed.

## Supplementary information

**Additional file 1: Figure S1.** Phylogenetic tree based on *cox*1 gene sequences of ticks. Numbers at each node indicate bootstrap values (only numbers > 70 are shown). The tree was mid-point rooted for clarity and the scale-bar represents the number of nucleotide substitutions per site. Representative strains herein were used to reconstruct the tree and marked by circles.

**Additional file 2: Figure S2.** Alignment of the representative TRAP amino acid sequences between partial vWF-like A and TSR regions in each haplotype identified in this study and others in the GenBank database. Partial vWF-like A and TSR regions were indicated by red and blue color, respectively.

## Data Availability

Data supporting the conclusions of this article are included within the article and its additional files. The *18S* rRNA, ITS, *TRAP* and *cytb* gene sequences generated in this study for phylogenetic analysis were submitted to the GenBank database under the accession numbers MN928814-MN928897.

## Abbreviations

rRNA: ribosomal RNA
PCR: polymerase chain reaction
*TRAP*: thrombospondin-related adhesive protein
ML: Maximum Likelihood
TR: tandem repeat
vWF-like A: von Willebrand Factor-like A
TSR: a thrombospondin type I repeat
CTD: cytoplasmic tail domain
ATV: atovaquone
