# Supplementary figures and images for: Molecular detection and genetic characteristics of Babesia gibsoni in dogs in Shaanxi Province, China
Source: Parasit Vectors. 2020 Jul 22;13:366. doi: 10.1186/s13071-020-04232-w (PMC7376908; doi:10.1186/s13071-020-04232-w)

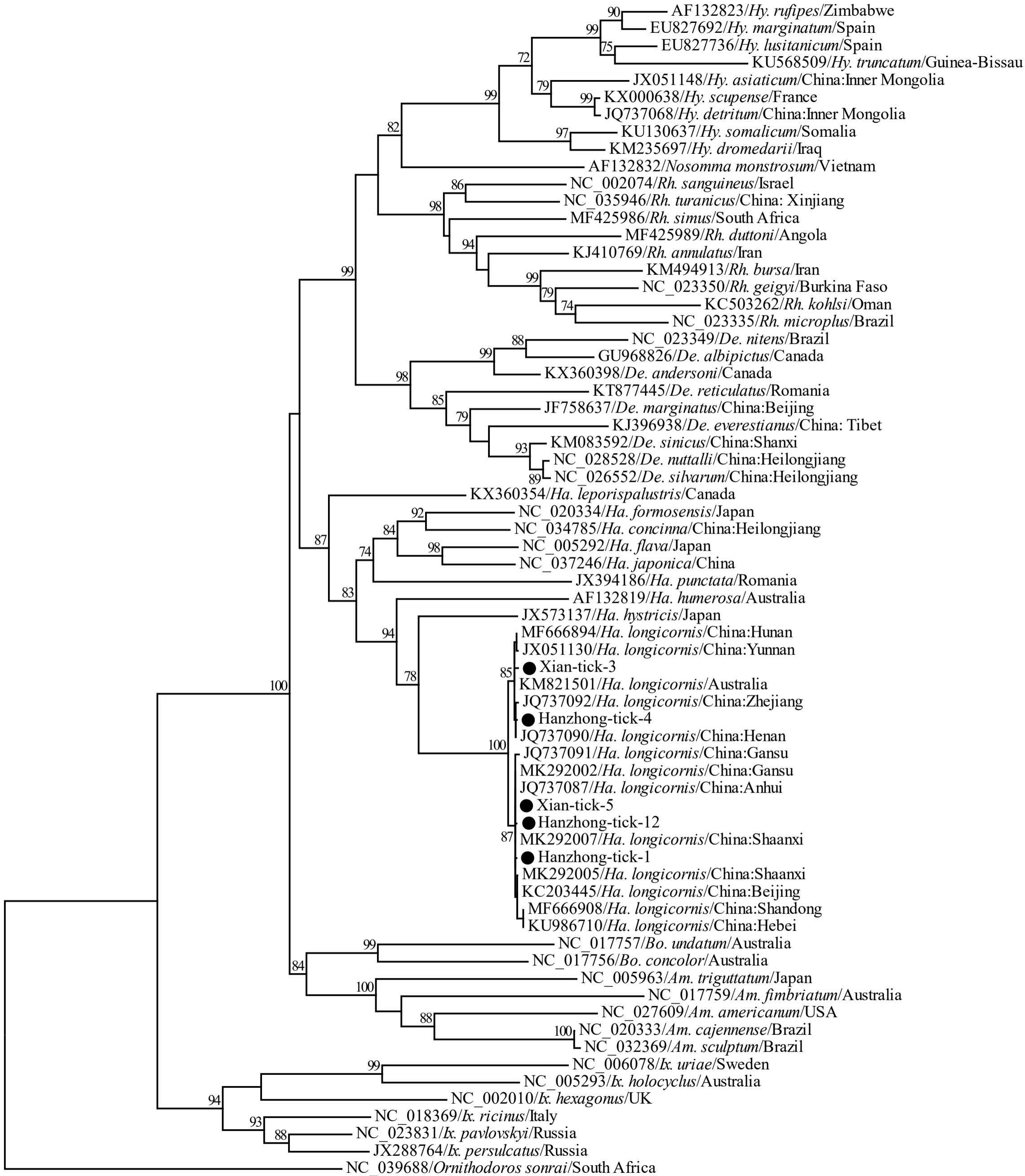

0.1

Supplement: Supplementary file 1 — Additional file 1: Figure S1. Phylogenetic tree based on cox1 gene sequences of ticks. Numbers at each node indicate bootstrap values (only numbers > 70 are shown). The tree was mid-point rooted for clarity and the scale-bar represents the number of nucleotide substitutions per site. Representative strains herein were used to reconstruct the tree and marked by circles. [file 13071_2020_4232_MOESM1_ESM.pdf]
