# Supplementary material for: Molecular detection and genetic characteristics of Babesia gibsoni in dogs in Shaanxi Province, China
Source: Parasit Vectors. 2020 Jul 22;13:366. doi: 10.1186/s13071-020-04232-w (PMC7376908; doi:10.1186/s13071-020-04232-w)
Supplement: Supplementary file 2 — Additional file 2: Figure S2. Alignment of the representative TRAP amino acid sequences between partial vWF-like A and TSR regions in each haplotype identified in this study and others in the GenBank database. Partial vWF-like A and TSR regions were indicated by red and blue color, respectively. [file 13071_2020_4232_MOESM2_ESM.pdf]

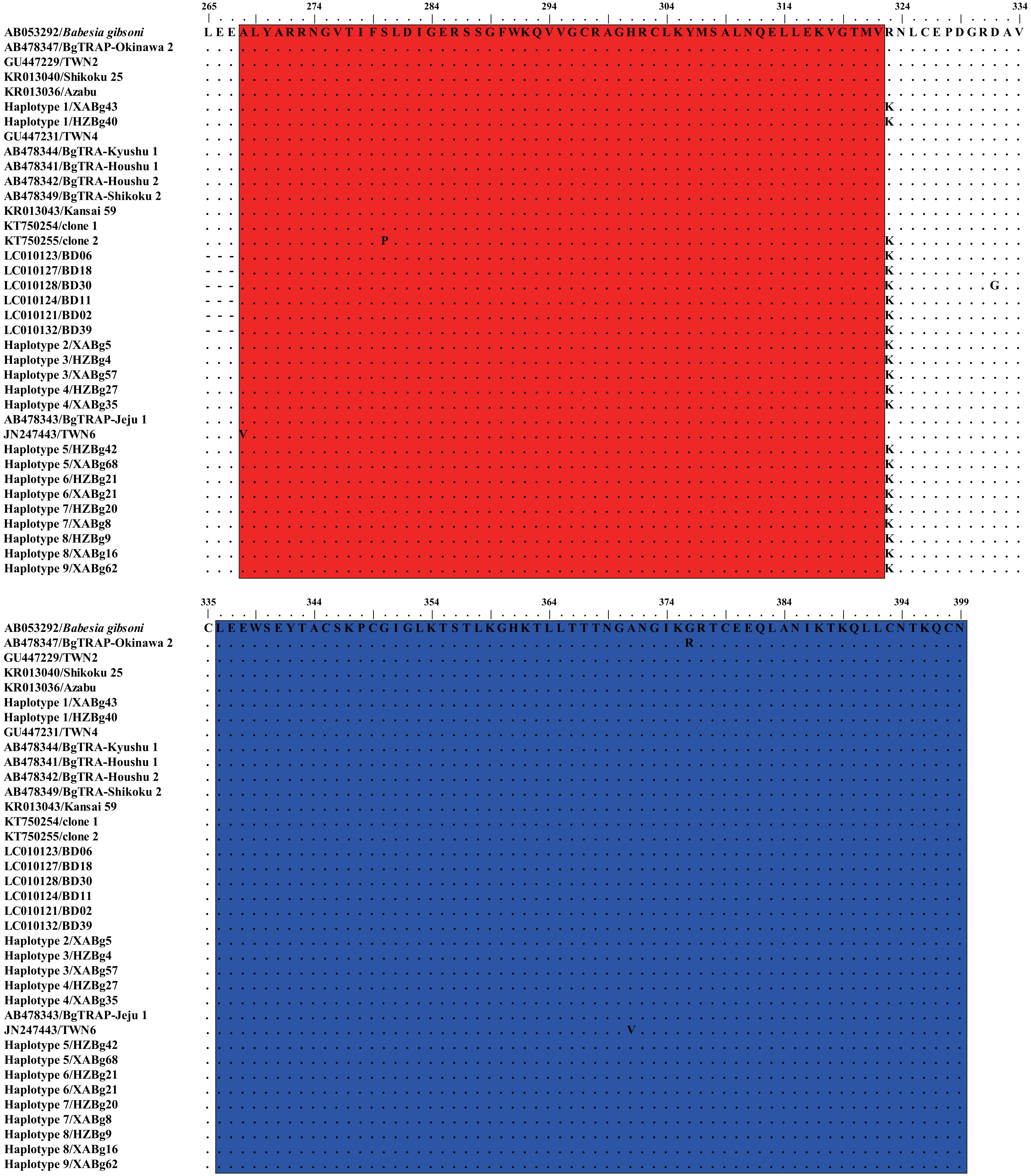
265 274 284 294 304 314 324 334
AB053292/Babesia gibsoni
L E E A L Y A R R N G V T I F S L D I G E R S S G F W K Q V V G C R A G H R C L K Y M S A L N Q E L L E K V G T M V R N L C E P D G R D A V
AB478347/BgTRAP-Okinawa 2
GU447229/TWN2
KR013040/Shikoku 25
KR013036/Azabu
Haplotype 1/XABg43
Haplotype 1/HZBg40
GU447231/TWN4
AB478344/BgTRA-Kyushu 1
AB478341/BgTRA-Houshu 1
AB478342/BgTRA-Houshu 2
AB478349/BgTRA-Shikoku 2
KR013043/Kansai 59
KT750254/clone 1
KT750255/clone 2
LC010123/BD06
LC010127/BD18
LC010128/BD30
LC010124/BD11
LC010121/BD02
LC010132/BD39
Haplotype 2/XABg5
Haplotype 3/HZBg4
Haplotype 3/XABg57
Haplotype 4/HZBg27
Haplotype 4/XABg35
AB478343/BgTRAP-Jeju 1
JN247443/TWN6
Haplotype 5/HZBg42
Haplotype 5/XABg68
Haplotype 6/HZBg21
Haplotype 6/XABg21
Haplotype 7/HZBg20
Haplotype 7/XABg8
Haplotype 8/HZBg9
Haplotype 8/XABg16
Haplotype 9/XABg62
335 344 354 364 374 384 394 399
C L E E W S E Y T A C S K P C G I G L K T S T L K G H K T L L T T T N G A N G I K G R T C E E Q L A N I K T K Q L L C N T K Q C N
